# Galactomannan inhibits *Trichinella spiralis* invasion of intestinal epithelium cells and enhances antibody-dependent cellular cytotoxicity related killing of larvae by driving macrophage polarization[Fn FN1]

**DOI:** 10.1051/parasite/2024002

**Published:** 2024-02-08

**Authors:** Ru Zhang, Yao Zhang, Shu Wei Yan, Yong Kang Cheng, Wen Wen Zheng, Shao Rong Long, Zhong Quan Wang, Jing Cui

**Affiliations:** Department of Parasitology, Medical College, Zhengzhou University Zhengzhou 450052 China

**Keywords:** *Trichinella spiralis*, Galactomannan, Invasion, Macrophage, ADCC

## Abstract

Previous studies have shown that recombinant *Trichinella spiralis* galectin (rTsgal) is characterized by a carbohydrate recognition domain sequence motif binding to beta-galactoside, and that rTsgal promotes larval invasion of intestinal epithelial cells. Galactomannan is an immunostimulatory polysaccharide composed of a mannan backbone with galactose residues. The aim of this study was to investigate whether galactomannan inhibits larval intrusion of intestinal epithelial cells and enhances antibody-dependent cellular cytotoxicity (ADCC), killing newborn larvae by polarizing macrophages to the M1 phenotype. The results showed that galactomannan specially binds to rTsgal, and abrogated rTsgal facilitation of larval invasion of intestinal epithelial cells. The results of qPCR, Western blotting, and flow cytometry showed that galactomannan and rTsgal activated macrophage M1 polarization, as demonstrated by high expression of iNOS (M1 marker) and M1 related genes (IL-1β, IL-6, and TNF-α), and increased CD86^+^ macrophages. Galactomannan and rTsgal also increased NO production. The killing ability of macrophage-mediated ADCC on larvae was also significantly enhanced in galactomannan- and rTsgal-treated macrophages. The results demonstrated that Tsgal may be considered a potential vaccine target molecule against *T. spiralis* invasion, and galactomannan may be a novel adjuvant therapeutic agent and potential vaccine adjuvant against *T. spiralis* infection.

## Abstract (fr)

Des études antérieures ont montré que la galectine recombinante de *Trichinella* *spiralis* (rTsgal) est caractérisée par un motif de séquence de domaines de reconnaissance des glucides se liant au bêta-galactoside, et que la rTsgal favorise l’invasion larvaire des cellules épithéliales intestinales. Le galactomannane est un polysaccharide immunostimulateur composé d’un squelette mannane avec des résidus galactose. Le but de cette étude était de déterminer si le galactomannane inhibe l’intrusion larvaire des cellules épithéliales intestinales et améliore la cytotoxicité cellulaire dépendante des anticorps (CCDA) tuant les larves nouvelles-nées en polarisant les macrophages au phénotype M1. Les résultats ont montré que le galactomannane se liait spécialement au rTsgal et supprimait la facilitation du rTsgal sur l’invasion larvaire des cellules épithéliales intestinales. Les résultats de la qPCR, du Western blot et de la cytométrie en flux ont montré que le galactomannane et le rTsgal activaient la polarisation des macrophages M1, comme le démontre la forte expression de l’iNOS (marqueur de M1) et des gènes liés à M1 (IL-1β, IL-6 et TNF-α), et l’augmentation des macrophages CD86^+^. Le galactomannane et le rTsgal ont également augmenté la production de NO. La capacité de destruction de la CCDA médiée par les macrophages sur les larves était également significativement améliorée dans les macrophages traités au galactomannane et au rTsgal. Les résultats ont démontré que Tsgal pourrait être considéré comme une molécule cible potentielle d’un vaccin contre l’invasion par *T. spiralis*, et que le galactomannane pourrait être un nouvel agent thérapeutique adjuvant et un adjuvant vaccinal potentiel contre l’infection à *T. spiralis*.

## Introduction

*Trichinella spiralis* (Owen, 1835) is a parasitic nematode, and its adult and larval worms lodge in a same host. The parasite has a complex life cycle. After being ingested, the muscle larvae (ML) in infected meat are liberated from the capsule under the action of digestive fluid. The ML develop into intestinal infectious larvae (IIL), following stimulation by bile and gut contents. The IIL invade the gut epithelia and undergo molting four times to develop into adult worms (AW) [40]. The pregnant female AWs produce the newborn larvae (NBL) which migrate to muscle tissues through the blood and lymphatic circulatory system. The NBL then invade skeletal muscles to develop into encapsulated ML to complete the lifecycle [12]. Humans acquire *Trichinella* infection by consuming raw or undercooked animal meat containing *Trichinella* infective larvae [43, 76]. Many outbreaks of human trichinellosis have been reported in certain countries [13, 57]; trichinellosis is still a major food-borne zoonosis with economic, social and public health impacts. Thus, new effective strategies are needed to prevent and control this important zoonotic disease.

Macrophages are widely distributed in various tissues of the organism. In parasite infection, macrophages are the most concentrated immune cells, which play an important role in hosts defending parasite infection and regulating immune response. After parasite infection, macrophages are differentiated into two subtypes during the process of host immune response, including classically activated macrophages (M1) and alternatively activated macrophages (M2) [27, 71]. M1 macrophages induce high expression of inducible nitric oxide synthase (iNOS), secrete inflammatory factors and chemokines, participate in immune response, and play the function of immune surveillance [26]. M2 macrophages can be activated by IL-4 and have high expression of Arginase-1 (Arg-1), which is involved in wound healing and tissue repair [3].

Polysaccharides are a class of natural polymers composed of carbohydrate monomers linked by glycosyl, which could bind with specific receptors in host cells to activate macrophages, lymphocytes, and natural killer cells, promoting the secretion of pro-inflammatory cytokines (TNF-α, IL-1β and IL-6), antibodies, complement, as well as increasing the production levels of nitric oxide (NO) and reactive oxygen species (ROS) [9]. These inflammatory mediators enhance the macrophage phagocytosis and effectively kill invaded parasites. Previous studies have shown that NO participates in macrophages-mediated killing to *Schistosoma*, *Plasmodium*, *Leishmania*, and *Toxoplasma* [2, 34, 60, 78]. Additionally, some researchers have also observed that NO effectively combats *T. spiralis* infection and reduces worm burdens [16]. Other studies have shown that macrophages directly killed and destroyed *T. spiralis* NBL by releasing NO and mediating antibody-dependent cellular cytotoxicity (ADCC) as a kind of main effector cells [15].

Galactomannan (GM) is an important polysaccharide that has diverse biological activities. The galactomannan derived from *Antrodia cinnamomea* and *Sesbania cannabina* plays an immune regulatory role by interacting with TLR4 receptors on macrophages [39, 55]. In parasite infection, α-lactose containing galactoside has been found to manipulate the galectin signal in mice [46]. A galactomannan (GALMAN-A) isolated from seeds of *Mimosa scabrella* and its oxovanadium complex have been shown to potentially activate macrophages to release potentially leishmanicidal mediators such as NO, IL-1β, and IL-6 [1]. Findings have suggested that galactomannan may activate macrophages and participate in immune modulation.

In previous research in our laboratory, a *T. spiralis* beta-galactoside-binding galectin (Tsgal; GenBank: XM_003381608.1) was identified. rTsgal is a tandem lectin protein containing two carbohydrate recognition domains (CRD), specifically binding to intestinal epithelial cells (IECs) and facilitating larval invasion of IECs [62, 64]. A *T. spiralis* type C lectin (TsCTL) is a surface and secretory protein highly expressed at the IIL stage, and rTsCTL facilitates IIL invasion of IECs, whereas anti-rTsCTL antibodies and mannose obviously suppressed IIL penetration. Although mannose reduced parasite burden, it also inhibited host humoral and cellular immune responses in *T. spiralis*-infected mice, suggesting that mannose plays an immunosuppressive role during *T. spiralis* infection [20, 21]. Therefore, it is necessary to identify a saccharide that has two functions for blocking larval invasion and enhancing host immune responses. Galactomannan, as a polysaccharide containing mannose and galactose, is likely to competitively suppress the binding and interaction between rTsgal and host cell receptors through CRD, and to impede larval invasion of IECs. Nevertheless, there are no reports in the literature on the biological activity and function of galactomannan during *T. spiralis* infection. In this study, the galactomannan effects on larval invasion of IECs were investigated in an *in vitro* invasion cell model. The immune modulatory effects of galactomannan and rTsgal on macrophages were also ascertained in RAW264.7 macrophages and mouse peritoneal macrophages.

## Materials and methods

### *Trichinella* species, cells, and experimental animals

*Trichinella spiralis* (ISS534) used in the current research was obtained from infected pigs of Henan Province and maintained in BALB/c mice [30]. The IECs were obtained from the small intestine of BALB/c mice in our laboratory [5]. Murine monocyte/macrophage cell line RAW264.7 was obtained from the Cell Bank of the Chinese Academy of Sciences, and the cells were incubated at 37 °C in 5% CO_2_ with Dulbecco’s modified Eagle medium (DMEM) containing 10% fetal bovine serum, 100 U/mL penicillin and 10 μg/mL streptomycin [10]. Female BALB/c mice at 4–6 weeks of age were purchased from the Experimental Animal Center of Zhengzhou University. All the mice were bred in individual ventilated cages under specific-pathogen-free (SPF) conditions.

### Ethics

The animal experiments in this study were performed under the principles of the National Guidelines for Experimental Animal Welfare of China. All animal experimental procedures were authorized by the Life Science Ethics Committee of Zhengzhou University (No. ZZUIRB GZR 2022-1317).

### Preparation of recombinant Tsgal (rTsgal) and anti-rTsgal serum

The complete sequence of the Tsgal gene (XM_003381608.1) was cloned, and recombinant expression plasmid pQE-80L/Tsgal was transferred to *Escherichia coli* BL21 (DE3) (Novagen, Glendale, CA, USA). rTsgal expression was induced by using 0.5 mM isopropyl β-D-thiogalactopyranoside (IPTG) at 37 °C for 6 h in our laboratory [62]. The rTsgal protein was purified using Ni-NTA-Sefinose resin (Sangon Biotech Co., Shanghai, China), and identified by SDS-PAGE and Western blotting analysis [47, 65].

Twenty 4–6-week-old BALB/c mice were subcutaneously immunized with 20 μg rTsgal emulsified with complete Freund’s adjuvant. After that, three boost immunizations were administered by 20 μg rTsgal emulsified with incomplete Freund’s adjuvant at a 2-week interval. After the last immunization, serum samples were collected and the antibody titer was measured by ELISA using rTsgal as coating antigen [17].

### Collection of different stage worms of *T. spiralis* and soluble somatic antigen preparation

Murine muscles infected with *T. spiralis* were artificially digested at 45 days post-infection (dpi) to obtain ML. The IIL were harvested from the small intestine at 6 h post-infection (hpi) [35, 42]. AWs were collected from infected mouse small intestines at 3 dpi and 6 dpi. NBL were collected from 6-day pregnant females cultured for 18 h [61]. The soluble somatic worm antigens from various *T. spiralis* stages (ML, IIL, AW and NBL) were prepared as previously described [23].

### Real-time quantitative PCR (qPCR)

Total RNAs of different *T. spiralis* stages (ML, IIL, AW and NBL) were extracted using Trizol reagent (Takara, Japan), and then reversely transcribed into cDNAs [36]. The transcription levels of Tsgal gene were ascertained by qPCR with specific primers (5′–TATTCGCAAGGGTGCGATGG–3′; 5′–ATCTTTCGCACGTTGATGGC–3′). The glyceraldehyde-3-phosphate dehydrogenase (GAPDH, GenBank: AF452239S) gene was used as an internal control, and there were no differences in GAPDH expression among different groups. The fold changes of Tsgal gene in diverse worm stages were calculated using the comparative Ct (2^−ΔΔCt^) method [25, 40]. Each experiment was carried out in triplicate.

Additionally, RAW264.7 and peritoneal macrophages (2 × 10^6^ cells/well) were seeded in 6-well plates and pretreated using 20 μg/mL rTsgal or 1.6% GM at 37 °C for 24 h. Expression of M1 (IL-1β, IL-6, and TNF-α) and M2 (IL-10, TGF-β) cytokines as well as M2 marker CD206 was also determined by qPCR. Primer sequences used for qPCR amplification are shown in Table 1 [7, 33].

**Table 1:** Specific primer sequences of macrophage markers and cytokines for qPCR.

Gene	Forward (5′–3′)	Reverse (5′–3′)
GAPDH	GGTTGTCTCCTGCGACTTCA	TGGTCCAGGGTTTCTTACTCC
IL-1β	TGGACCTTCCAGGATGAGGACA	GTTCATCTCGGAGCCTGTAGTG
IL-6	TACCACTTCACAAGTCGGAGGC	CTGCAAGTGCATCATCGTTGTTC
TNF-α	GGTGCCTATGTCTCAGCCTCTT	GCCATAGAACTGATGAGAGGGAG
IL-10	CGGGAAGACAATAACTGCACCC	CGGTTAGCAGTATGTTGTCCAGC
TGF-β	TGATACGCCTGAGTGGCTGTCT	CACAAGAGCAGTGAGCGCTGAA
CD206	GTTCACCTGGAGTGATGGTTCTC	AGGACATGCCAGGGTCACCTTT

### Western blot analysis

Western blot analysis was performed according to previous studies [19]. Soluble somatic antigens of different *T. spiralis* stages were separated by SDS-PAGE and then transferred to polyvinylidene difluoride (PVDF) membranes (Millipore, Burlington, MA, USA), blocked with 5% skimmed milk at 37 °C for 2 h. The membrane was cut into strips, and incubated with 1:200 dilutions of anti-Tsgal serum at 37 °C for 1 h. After being washed with TBST, the strips were further incubated with HRP-conjugated goat anti-mouse IgG (1:5000; Southern Biotech, Birmingham, AL, USA) at 37 °C for 1 h, and visualized by Omni-ECL^Tm^ reagents (Epizyme, Shanghai, China) using chemiluminescent gel imaging system (Tanon 5200, Shanghai, China). The relative intensities of each protein band were analyzed using ImageJ software (National Institutes of Health, Bethesda, MD, USA) [17, 50, 59].

### ELISA determination of binding of galactomannan and rTsgal

Binding of GM with rTsgal was detected by ELISA [41]. The GM (99.00% purity) used in this study was a commercial chemical reagent (Kangda Biotech., Guangzhou, China). Briefly, a plate was coated with diluted GM (0.25, 0.5, 1, 2 and 4%) at 4 °C overnight. After being blocked with 5% skim milk and washed using PBST, the plate was incubated with various concentrations of rTsgal (0.25, 0.5, 1, 2 and 4 μg/mL) at 37 °C for 1 h. Following washing, the plates were probed with anti-rTsgal serum (1:100) at 37 °C for 1 h, and then incubated with HRP-conjugated goat anti-mouse IgG (1:10,000; Southern Biotech). The plate was colored with o-phenylenediamine (OPD, Sigma, Saint Louis, MO, USA), and absorbance at 492 nm was measured by a multi-mode reader (SpectraMax i3X; Molecular Devices, San Jose, CA, USA) [47, 77].

### The *in vitro* larval invasive test

To analyse the role of rTsgal on larval intrusion of IECs, an *in vitro* larval invasion test was conducted as previous described [24, 37]. Briefly, ML were first activated into the IIL upon exposure to 5% swine bile for 2 h at 37 °C, and then added to semisolid medium (e.g., serum-free DMEM containing 1.75% agarose) with different doses of rTsgal (5, 10, 15 and 20 μg/mL). After culture at 5% CO_2_ at 37 °C for 2 h, larval intrusion into the monolayer was observed under microscopy. The invaded larvae were mobile and migrated within the monolayer, leaving a clear migratory trace, whereas non-invaded larvae were coiled on the monolayer surface [70].

To evaluate the GM effects on larval invasion, GM was used in the *in vitro* larval invasion test. The IILs were first pre-incubated with different concentrations of GM (0.25, 0.5, 1, 2 and 4%) at 37 °C for 2 h and then added to semisolid medium. After culturing at 5% CO_2_ at 37 °C for 2 h, larval penetration into IECs was examined by microscopy. To further investigate whether GM inhibits and abrogates the rTsgal facilitative role on larval invasion of IECs, 20 μg/mL of rTsgal was first pre-incubated with various doses of GM for 2 h, and then mixed with IILs, and the mixture containing GM-pretreated rTsgal and IILs was added to the cell monolayer. After culture at 5% CO_2_ at 37 °C for 2 h, larval intrusion of IECs was examined under microscopy [65]. The larvae invasion rate was defined as the percentage of invaded larvae to the number of total larvae observed in each assay. The promotion rate was calculated by the following formulas: promotion (%) = (mean invasion rate of experimental group – mean invasion rate of control group) × 100%. The inhibition rate was calculated by the following formulas: inhibition (%) =  (mean invasion rate of control group – mean invasion rate of experimental group) × 100% [21].

### Cell counting kit-8 (CCK-8) assay

The effect of rTsgal and GM on RAW264.7 macrophage cellular viability was ascertained by CCK-8 assay (Epizyme Biotech, Shanghai, China) [58, 68]. Briefly, the cells were seeded in 96-well plates at a density of 1 × 10^5^ cells and cultured in high glucose Dulbecco’s modified Eagle medium (DMEM; Servicebio, Wuhan, China) with 100 U/mL penicillin, 100 μg/mL streptomycin, and 10% fetal bovine serum (FBS; Gibco, Waltham, MA, USA). rTsgal and GM were added to the medium and co-cultured with the macrophages for 48 h. Then, 10 μL CCK-8 solutions were added to each well of the culture plate and incubated for 2 h. The absorbance was measured at 450 nm by a multi-mode reader.

### Preparation and *in vitro* stimulation of murine peritoneal macrophages

Murine peritoneal macrophages were prepared as previously described [70]. Briefly, mice were euthanized and soaked in 75% alcohol for 3–5 s. The mice were abdominally injected with 10 mL of pre-chilled PBS, and the peritoneal wall was simultaneously pressed for 10 min. Under sterile conditions, the peritoneal fluid was aspirated and centrifuged at 500×*g* for 10 min at 4 °C. Peritoneal exudate cells (PEC) were adjusted to 2 × 10^6^ cells/mL with complete DMEM medium. After incubation at 37 °C and in 5% CO_2_ for 4 h, the cells were washed three times with DMEM to remove unattached cells, and the purified peritoneal macrophages were obtained [4].

RAW264.7 and peritoneal macrophages (2 × 10^6^ cells/well) were cultivated in the presence of various stimulating factors. rTsgal (20 μg/mL) and GM (1.6%) were added to the medium, lipopolysaccharide (LPS, 500 ng/mL) and IL-4 (20 ng/mL) were used as macrophage M1/M2 polarization positive control, respectively and DMEM as a negative control. After incubation at 37 °C in 5% CO_2_ for diverse times, cells were washed with PBS and harvested for qPCR, Western blot, and flow cytometry analysis [18].

### Assay of nitric oxide (NO)

NO is an important inflammatory mediator, serving as a pleiotropic regulator in different biological pathways to defend against causative agents or foreign substances [54]. Macrophage NO production was assessed by measuring the accumulated nitrite released into culture media based on the Griess reaction [53]. Briefly, RAW 264.7 cells (1 × 10^5^ cells/well) were seeded in 96-well plates and incubated for 24 h. After renewal of the medium, cells were incubated in culture media supplemented with rTsgal (20 μg/mL) and GM (1.6%). LPS (500 ng/mL) or IL-4 (20 ng/mL) were used as M1/M2 positive control, respectively and DMEM was used as negative control. After stimulation for 24 h, NO production in the culture media was determined using an NO assay kit (Beyotime, Shanghai, China) based on the Griess reaction. After incubation at room temperature for 5 min, the solution’s absorbance at 540 nm was measured using a multi-mode reader. The standard curve was drawn according to different concentrations of NaNO_2_. All experiments were performed in triplicate [56].

### Determination of rTsgal and GM regulation on macrophage polarization

To investigate the regulation of rTsgal and GM on macrophage polarization, the RAW264.7 and murine peritoneal macrophages were used for the *in vitro* stimulation of M1 and M2 phenotypes. The cells (2 × 10^6^ cells/well) were stimulated using 20 μg/mL rTsgal or 1.6% GM at 37 °C for 24 h, soluble cell proteins were extracted using ice-cold cell lysis buffer (Beyotime, Shanghai, China) containing 1 mM phenylmethylsulfonyl fluoride (PMSF). Cell protein concentration was measured using a BCA assay kit (Solarbio, Beijing, China) [24, 69]. The expression level of M1 (iNOS)/M2 (Arg1) marker proteins in macrophages was then detected by Western blotting. Rabbit anti-iNOS antibody (1:1000; Abcam, Shanghai, China), mouse anti-Arg1 antibody (1:1000; Proteintech, Rosemont, IL, USA) and rat anti-tubulin antibody (1:5000; Abcam) were used as the primary antibodies; HRP-labeled goat anti-rabbit IgG, goat anti-mouse and goat anti-rat IgG (1:10000; Southern Biotech) were used as the second antibodies [32, 59].

### Flow cytometry staining

RAW264.7 and peritoneal macrophages (2 × 10^6^ cells/well) were pretreated with 20 μg/mL rTsgal or 1.6% GM at 37 °C for 24 h. The cell was pelleted by centrifugation at 500×*g*, 4 °C for 10 min, and the supernatant was discarded. Cell pellets were then resuspended in Fluorescence-Activated Cell Sorting (FACS) buffer (PBS, 0.1% BSA and 0.5 mM EDTA) for staining. Cells were blocked with anti-mouse CD16/CD32 antibody (mouse Fc blocker, 1:100; BD Biosciences, Franklin Lakes, NJ, USA) for 20 min on ice for flow cytometry. Cells were stained with antibodies against V450-F4/80 (eBioscience, San Diego, CA, USA), PerCP-Cyanine5.5-CD11b (eBioscience), PE-CD86 (eBioscience) and APC-CD206 (Biolegend San Diego, CA, USA). Subsequently, cells were washed, resuspended in PBS, and detected by flow cytometry (BD Biosciences). Dead cells and doublets were excluded from all analyses. Data were further analyzed using FlowJo software (Becton Dickinson and Company, Portland, OR, USA) [51, 63].

### Antibody-dependent cell-mediated cytotoxicity (ADCC) assay

The macrophage killing the NBL through ADCC was performed as previously reported [72]. Murine peritoneal macrophages (1 × 10^5^ cells/well) were seeded in 96-well plates and pre-incubated with various stimulators (DMEM, 1.6% GM, 500 ng/mL LPS, 20 ng/mL IL-4 and 20 μg/mL rTsgal) for 24 h. After renewal of the medium, 50 NBL were added to the medium containing 1:100 dilutions of *T. spiralis*-infected mouse serum and incubated at 37 °C for 72 h, DMEM medium-free serum was used as a negative control. After the NBL were cultured with various groups of macrophages and infection serum, larval viability was assessed based on larval morphology and activity. Living NBL were active and mobile, whereas dead NBL were inactive and straight. Cytotoxicity was defined as the percentage of dead NBL or NBL with adherent macrophages to the number of total larvae observed in each assay [4, 22].

### Statistical analysis

GraphPad Prism 9.0 and SPSS21.0 were used in this study to analyze experimental data, and the data are shown as mean ± standard deviation (SD). One-way ANOVA, linear regression, and chi-square tests were used to determine the statistical differences and correlation. *P* < 0.05 was defined as statistical significance.

## Results

### Transcription and expression of Tsgal at diverse stages of *T. spiralis*

**Figure 1:**
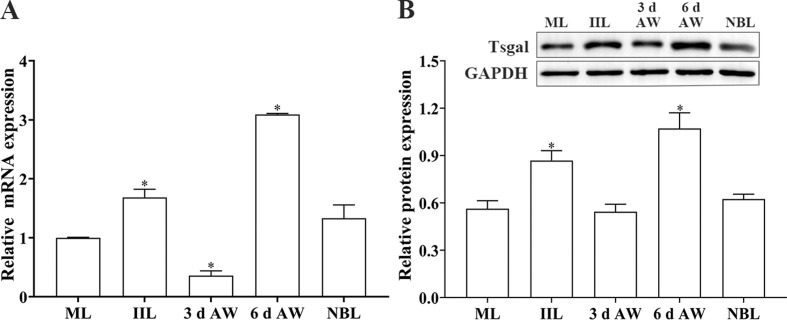
*Tsgal transcription and expression levels in diverse T. spiralis phases*. **A**: qPCR analysis of Tsgal transcription in different *T. spiralis* phases. The relative Tsgal mRNA expression level at IIL and 6 d AW phases was evidently higher than the other worm stages. **B:** Western blot analysis of the Tsgal expression level in different *T. spiralis* phases. Tsgal expression level at IIL and 6 d AW phases was also significantly higher than other worm phases. Asterisks indicate a statistically significant difference compared to the ML stage (*p < 0.05).

Transcription levels of Tsgal at diverse stages of *T. spiralis* were ascertained by qPCR. The results showed that the Tsgal transcription levels in the IIL and 6 d AW stages were significantly higher than the other worm stages (*F* = 68.11, *p* = 0.019) (Fig. 1A). Moreover, the Tsgal protein expression level in the IIL and 6 d AW stages was also higher than the other stages (*F* = 13.601, *p* = 0.021) (Fig. 1B). The results further suggested that Tsgal is an important intestinal *T. spiralis* invasion-related protein.

### Binding of GM with rTsgal detected by ELISA

**Figure 2:**
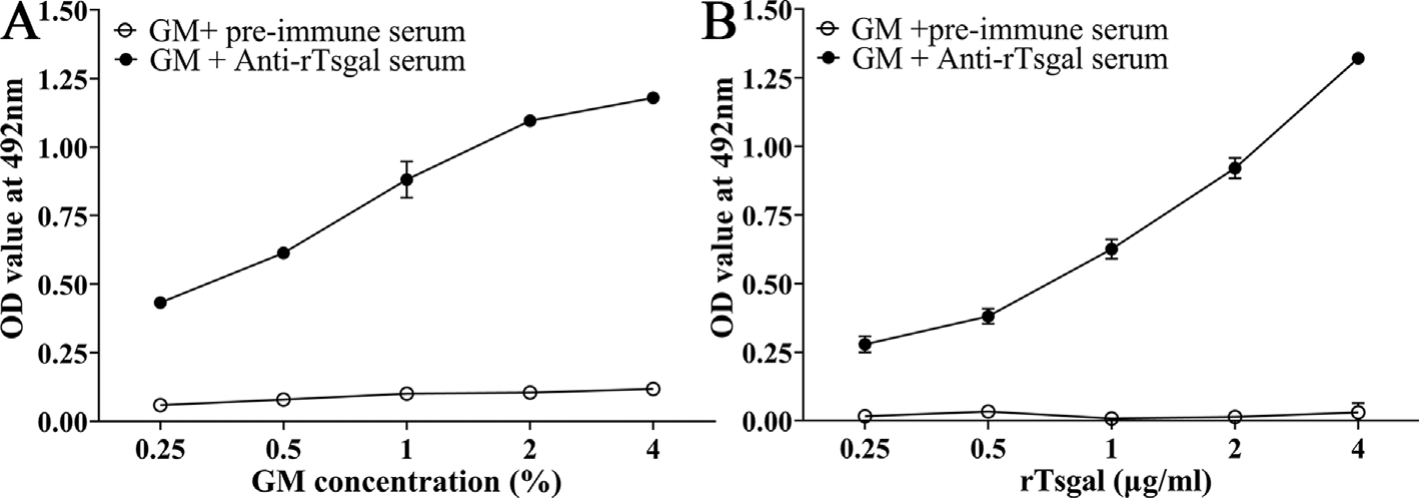
*Binding of GM and rTsgal determined by ELISA*. **A**: Different coating concentration of GM incubated with 5 μg/mL rTsgal. **B:** 1% GM was incubated with diverse doses of rTsgal. The plates were probed by diverse sera (pre-immune serum and anti-rTsgal serum).

The binding between GM and rTsgal was assessed by ELISA, and the results showed evident binding between GM and rTsgal. The binding capacity was dose-dependent of GM (*r*_GM_ = 0.875, *p *< 0.0001) and exhibited an elevating trend along with increasing GM dose (*F* = 209.156, *p *< 0.0001) (Fig. 2A). The binding capacity also was rTsgal dose-dependent (*r*_rTsgal_ = 0.982, *p *< 0.0001) and possessed a rising trend with increasing rTsgal dose (*F* = 409.259, *p *< 0.0001) (Fig. 2B). The results indicated that GM can bind to rTsgal, and the binding might suppress the rTsgal promoting role on larval intrusion of IECs.

### GM suppressed larval invasion of IECs

**Figure 3:**
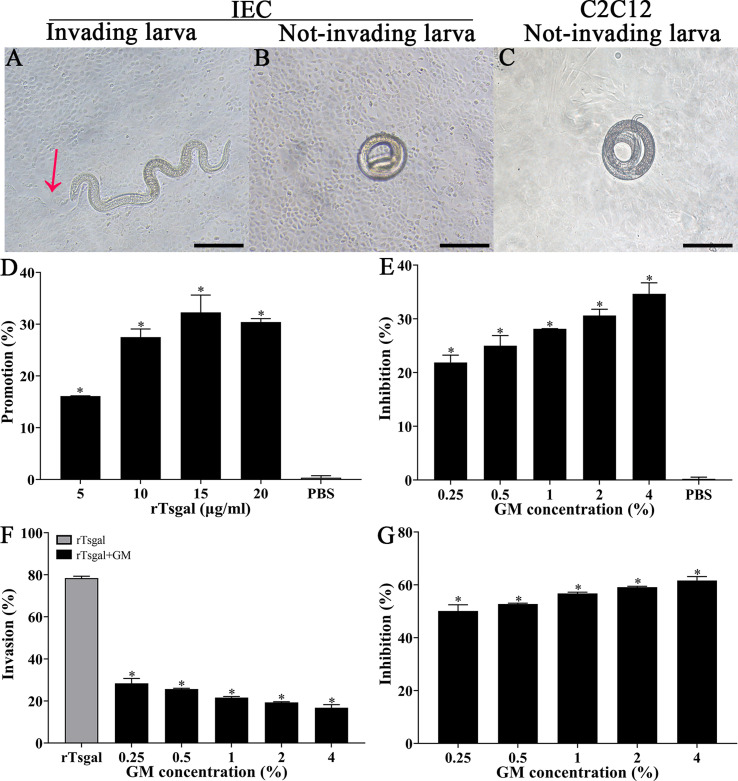
*GM suppressed larval invasion and abrogated the rTsgal promoting role on the invasion*. **A:** The invading larvae were mobile and migratory in the monolayer (the red arrow shows migratory trace). **B** and **C:** Non-invading worms were coiled on the surface of the IECs (**B**) and C2C12 cells (**C**). **D:** rTsgal accelerated IIL invasion into IECs. **E:** GM inhibited the IIL invasion of IECs in a GM dose-dependent manner. **F** and **G:** GM inhibited and abrogated the rTsgal facilitation effect on IIL invasion of IECs. Scale bars: 100 μm. * *p *< 0.05 compared to the PBS group (**D** and **E**) or only rTsgal groups (**F** and **G**).

To investigate the effect of GM on larval invasion of IECs, an *in vitro* invasive test was performed. The results showed that the invading larvae left a clear migratory trace (red arrow) (Fig. 3A), and not-invading larvae were spirally coiled on the surface of IECs and C2C12 monolayer (Fig. 3B, C). After the medium was supplemented with rTsgal, and cultured 2 h, rTsgal significantly accelerated larval intrusion in a rTsgal dose-dependent manner (*r* = 0.814, *p *< 0.05) and exhibited an increasing trend with increasing rTsgal dose (*F* = 14.788, *p *< 0.05) (Fig. 3D).

After IIL were first pre-incubated with various dilutions of GM at 37 °C for 2 h, the GM-treated IIL was added onto the IEC monolayer and co-cultured for 2 h, and larval invasion was significantly suppressed. The suppression of larval invasion of IECs by GM (0.25, 0.5, 1, 2 and 4%) was 21.88, 24.99, 28.14, 30.61 and 34.63%, respectively compared to the PBS group (*χ^2^*_0.25%_ = 6.139, *p *< 0.05; *χ^2^*_0.5%_ = 7.118, *χ^2^*_1%_ = 9.223, *p *< 0.001; *χ^2^*_2%_ = 13.403, *χ^2^*_4%_ = 14.338, *p *< 0.0001) (Fig. 3E). The suppression had a correlation with the GM doses (*r* = 0.970, *p *< 0.0001) and exhibited an elevating trend with the increase in GM dose (*F* = 21.995, *p *< 0.01). The results showed that GM evidently inhibited the IIL invasion of IECs and suggested that the GM pre-incubated IILs might result in the binding of GM with the Tsgal CRD, which impeded the binding and interaction between the Tsgal CRD in the IILs and ligand in IECs, consequently inhibited larval invasion.

To further verify whether GM abrogates the rTsgal facilitative effect on larval intrusion of IECs *in vitro*, 20 μg/mL rTsgal was pre-incubated with different doses of GM (0.25, 0.5, 1, 2 and 4%) at 37 °C for 2 h, and then, the IILs mixed with GM-treated rTsgal were added onto the IEC monolayer and co-cultured for 2 h. The results showed that GM evidently inhibited and abrogated the rTsgal facilitative effect on larval invasion of IECs compared to the only rTsgal group. The inhibition rate of 0.25, 0.5, 1, 2 and 4% GM on larval intrusion was 50.07, 52.71, 56.77, 59.12 and 61.66%, respectively (*χ^2^*_0.25%_ = 24.167, *χ^2^*_0.5%_ = 27.114, *χ^2^*_1%_ = 30.265, *χ^2^*_2%_ = 29.977, *χ^2^*_4%_ = 37.746, *p *< 0.0001) (Figs. 3F, 3G). The GM suppression role also had a correlation with the GM dose (*r* = 0.973, *p *< 0.0001) and exhibited an elevating trend with the increase of the GM dose (*F* = 27.185, *p *< 0.01). The results suggested that GM pre-incubated with rTsgal might suppress the binding of the rTsgal CRD with ligand in IECs, thereby abrogating the rTsgal promoting role on IEC invasion.

### Effect of rTsgal and GM on RAW264.7 macrophage viability

**Figure 4:**
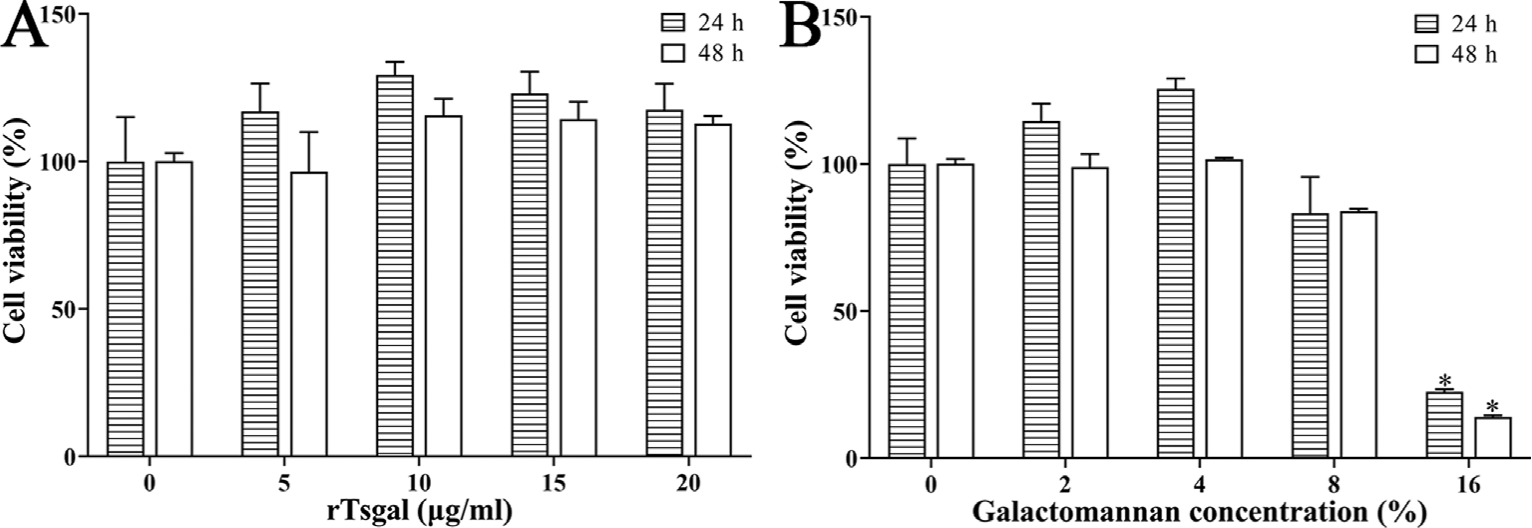
*Effect of rTsgal and GM on the viability of RAW264.7 cells*. rTsgal (5, 10, 15 and 20 μg/mL) and GM (2, 4, 8 and 16%) were co-incubated with RAW264.7 cells for 24 and 48 h, and cell viability was determined by CCK-8 assayed. Cell viability = (OD values of test group – OD values of blank control)/(OD values of PBS group – OD values of blank control) × 100%. *p < 0.05 compared to the PBS group.

To investigate the effect of rTsgal and GM on RAW264.7 cell viability, cells were treated with different concentrations of rTsgal (5, 10, 15 and 20 μg/mL) or GM (2, 4, 8 and 16%) for 24 and 48 h, and cellular viability was assessed by CCK-8 assay. The results showed that 5–20 μg/mL rTsgal had no obvious cytotoxicity on RAW264.7 macrophage viability after incubation for 24 and 48 h. However, 16% GM obviously decreased cell viability at 24 and 48 h compared to the PBS group (*F*_24h_ = 29.176, *F*_48h_ = 237.678*, p *< 0.001) (Fig. 4). Therefore, 20 μg/mL rTsgal and 1.6% GM were selected to be used in the following experiment.

### The effect of rTsgal and GM on macrophage NO production

**Figure 5:**
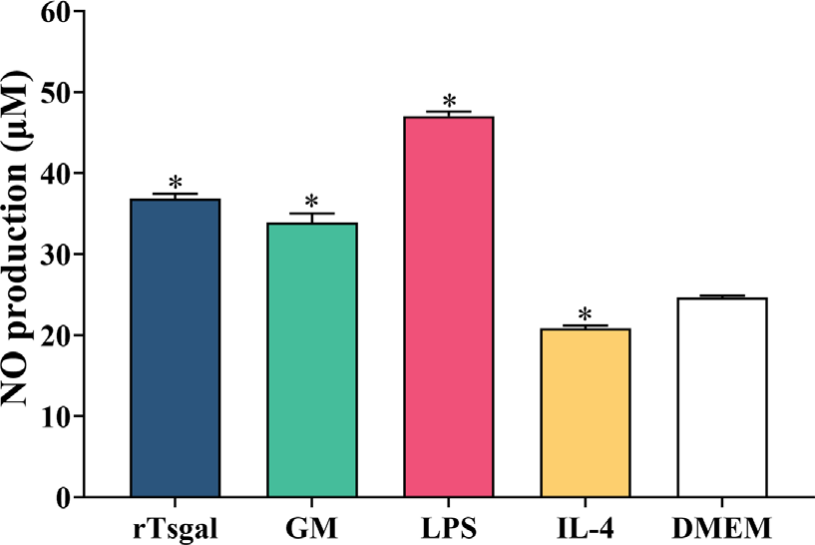
*Effects of rTsgal and GM on the production of NO in supernatant of cultured RAW264.7 cells.* Cells were treated with rTsgal and GM for 24 h. Each sample had three replicates, and statistical significance was analyzed using ANOVA. *p < 0.001 compared to the DMEM group.

To assess the effect of rTsgal and GM on NO production of RAW264.7 macrophages, Griess reaction was performed. The results revealed that rTsgal and GM significantly increased NO production (*F* = 808.115, *p *< 0.001) (Fig. 5). Moreover, the NO output in the rTsgal and GM group was obviously higher than the medium DMEM control group. Nevertheless, NO output of the M2 positive control (IL-4) group was decreased relative to the DMEM group (*F* = 808.115, *p *< 0.001). The results suggested that rTsgal and GM induced and activated macrophages M1 polarization.

### rTsgal and GM promote macrophage M1 polarization

**Figure 6:**
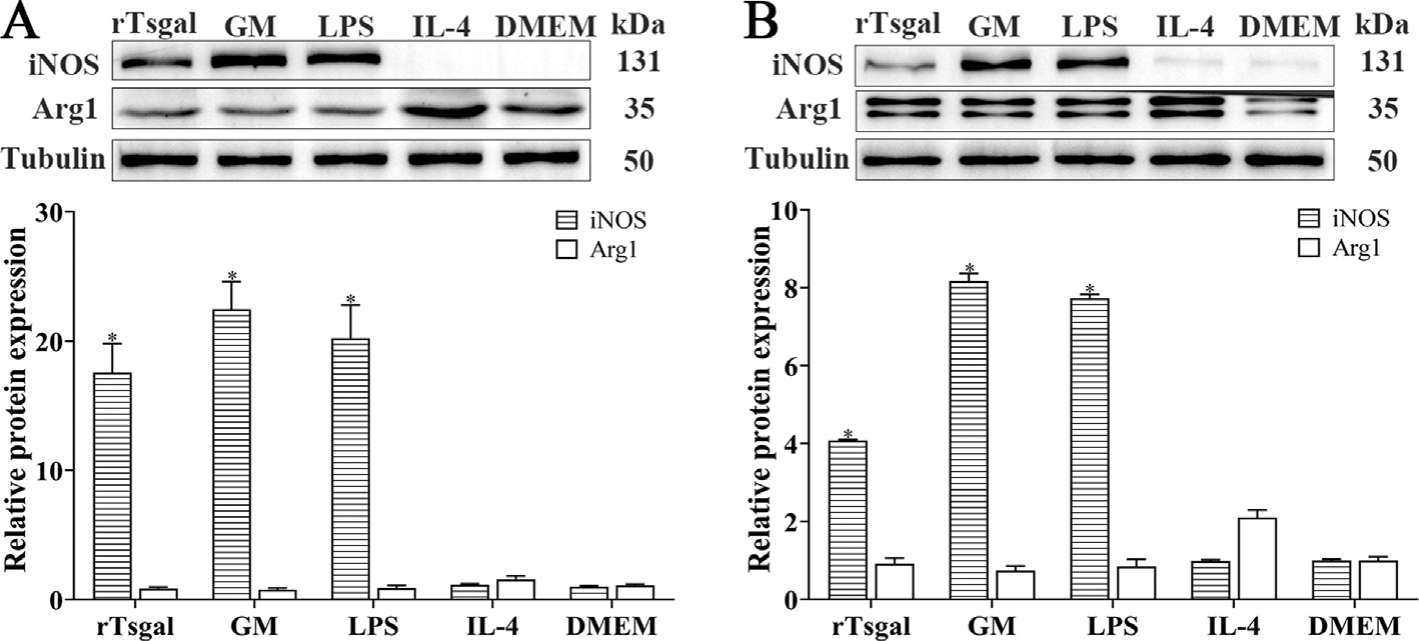
*Expression level of M1/M2 marker protein in RAW264.7 cells (****A****) and murine peritoneal macrophages (****B****)*. The expression of iNOS and Arg1 in macrophages was analyzed on Western blotting after the cells were incubated with 20 μg/mL rTsgal or 1.6% GM at 37 °C for 24 h. Tubulin served as the internal control. Each experiment was performed in triplicate. Arg1 expressed in RAW264.7 macrophages had a monomer band (**A**); whereas Arg1 expressed in murine peritoneal macrophages had a dimer band (**B**). *p < 0.0001 compared to the DMEM group.

The effect of rTsgal and GM on macrophage polarization was investigated by Western blotting analysis. The results showed that after RAW264.7 macrophages were stimulated by rTsgal and GM, the expression level of M1 marker iNOS was significantly increased relative to the DMEM control group (*F* = 34.755, *p *< 0.0001), but the protein expression level of Arg-1 (M2 marker) was not evidently changed compared to the DMEM group (Fig. 6A). To further ascertain the effect of rTsgal and GM on macrophage polarization, murine peritoneal macrophages were also stimulated by rTsgal and GM. The expression level of iNOS was also obviously increased in the rTsgal and GM group, when compared to the DMEM control group (*F*_iNOS_ = 1210.2, *p *< 0.0001) (Fig. 6B). The results suggest that rTsgal and GM drove macrophages M1 polarization.

### rTsgal and GM promote the production of IL-1β, IL-6, and TNF-α in macrophages

**Figure 7:**
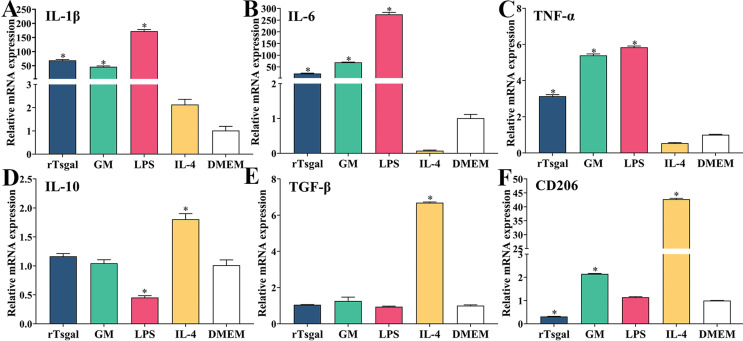
*mRNA expression level of M1/M2 genes in RAW264.7 macrophages*. The mRNA expression of M1/M2 cytokines in RAW264.7 macrophages treated with rTsgal or GM was analyzed by qPCR. After treatment with 20 μg/mL rTsgal or 1.6% GM at 37 °C for 24 h, the transcription level of M1 cytokines (IL-1β, IL-6 and TNF-α) was clearly increased in RAW264.7 macrophages, whereas the transcription level of M2 cytokines (IL-10, TGF-β) and surface marker CD206 was not evidently changed. The mRNA expression levels of the cytokines were calculated with the Ct ^(2−ΔΔCt)^ method. GAPDH was utilized as an internal control. *p < 0.0001 compared to the DMEM group.

**Figure 8:**
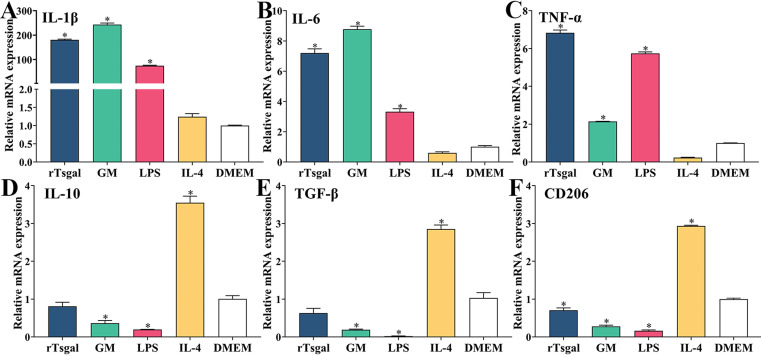
*mRNA expression level of M1/M2 cytokines in murine peritoneal macrophages*. The mRNA expression of M1 cytokines (IL-1β, IL-6, and TNF-α) and M2 cytokines (IL-10, TGF-β) and marker CD206 in mouse peritoneal macrophages was measured by qPCR. The relative mRNA expression was calculated with the Ct ^(2−ΔΔCt)^ method. GAPDH was utilized as an internal control. *p < 0.0001 compared to the DMEM group.

In order to investigate the role of rTsgal and GM on macrophage polarization, RAW264.7 and peritoneal macrophages were incubated with 20 μg/mL rTsgal or 1.6% GM at 37 °C for 24 h, and the mRNA expression level of M1/M2-related genes was analyzed by qPCR. The results revealed that the mRNA expression level of M1 cytokines (IL-1β, IL-6, and TNF-α) in rTsgal and GM-treated RAW264.7 cells was obviously increased (*F*_IL-1β_ = 1474.531, *F*_IL-6_ = 4249.05, *F*_TNF-α_ = 2367.43, *p *< 0.0001), while the mRNA expression level of M2 cytokines (IL-10, TGF-β) was not significantly changed in the rTsgal and GM-treated group compared to the DMEM group (Fig. 7). Furthermore, after being treated with rTsgal and GM, mRNA expression levels of M2-related genes in murine peritoneal macrophages were not obviously increased when compared to the DMEM group; however, the mRNA expression of M1 genes was significantly increased (*F*_IL-1β_ = 1131.354, *F*_IL-6_ = 404.778, *F*_TNF-α_ = 1502.526, *p *< 0.0001) (Fig. 8). The results demonstrated that rTsgal and GM induced activation of M1 phenotypic macrophages and evidently induced mRNA expression levels of M1 cytokines (IL-1β, IL-6, and TNF-α).

### rTsgal and GM induced high expression of M1 maker CD86 in macrophages

**Figure 9:**
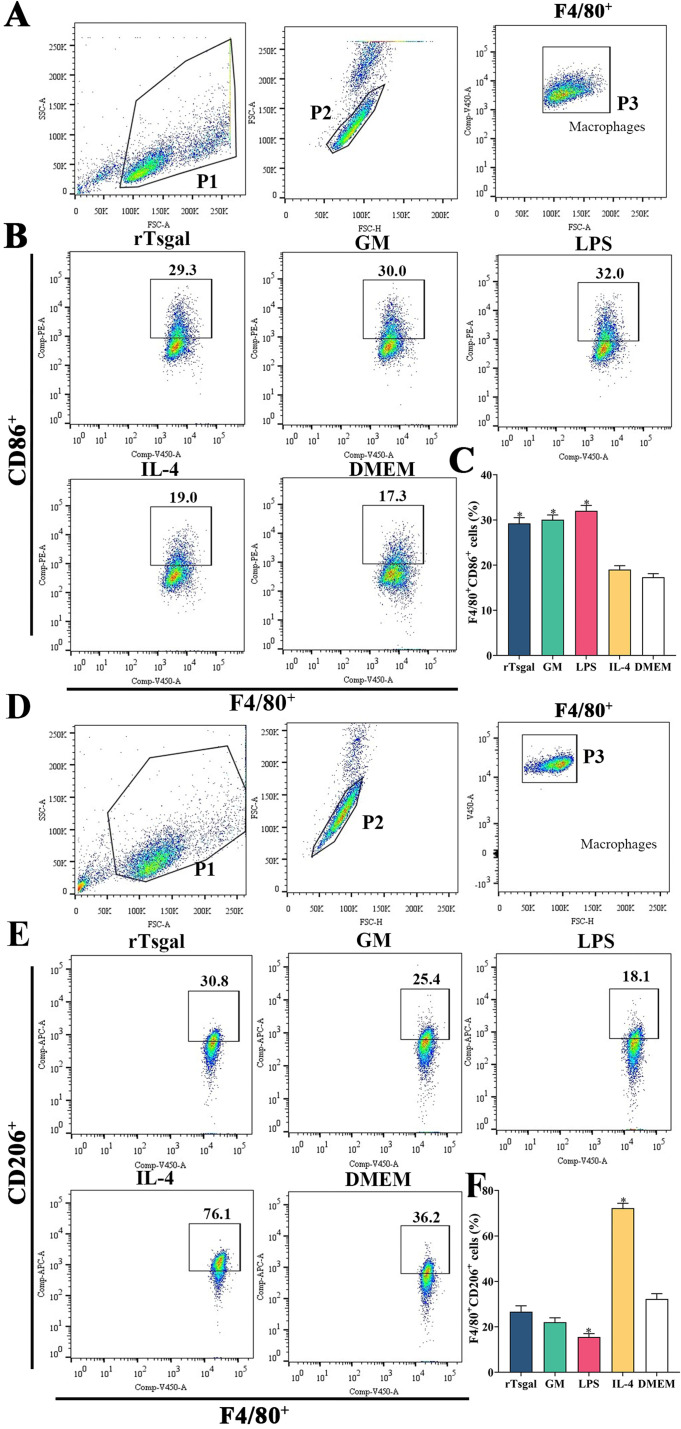
*Expression of M1/M2 markers in RAW264.7 cells assayed by flow cytometry*. The RAW264.7 macrophages with F4/80^+^CD86^+^ phenotype was identified as M1 macrophages, and those with a F4/80^+^CD206^+^ phenotype was designated as M2 macrophages. **A:** Gating strategy of macrophages. The cell population was analyzed in the P1 gate, and single cell in the P2 was circled using forward scatter-area (FSC-A) and forward scatter-height (FSC-H). Macrophages were identified using F4/80 antibody in the P3 gate. **B:** Gating strategy of M1 macrophages. Black box displayed the F4/80^+^CD86^+ ^M1 cells. **C:** The percentage of CD86 positive cells was shown in the bar graph. **D:** Gating strategy of macrophages was the same as (A). **E:** Black box displayed F4/80^+^CD206^+ ^M2 macrophages. **F:** The percentage of CD206 positive cells is shown in the bar graph. *p < 0.01 compared to the DMEM group.

**Figure 10:**
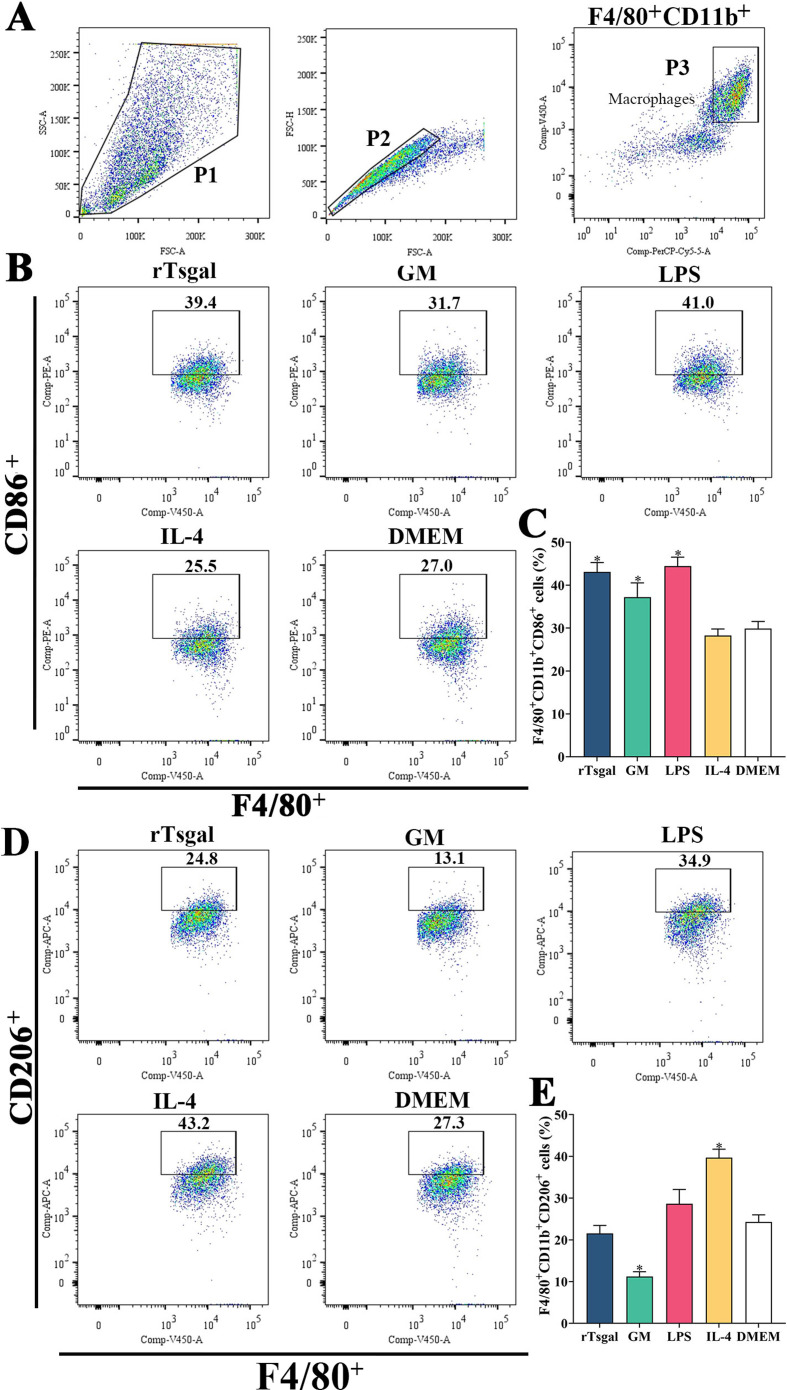
*Expression of M1/M2 markers in mouse peritoneal macrophages determined by flow cytometry*. The peritoneal macrophages with F4/80^+^CD11b^+^CD86^+^ phenotype were identified as M1 macrophages, and those with a F4/80^+^CD11b^+^CD206^+^ phenotype were designated as M2 macrophages. **A:** Gating strategy of macrophages. The cell population was analyzed in the P1 gate, and single cell in the P2 was circled using forward scatter-area (FSC-A) and forward scatter-height (FSC-H). Macrophages were identified using F4/80 and CD11b antibodies in the P3. **B:** Black box displays M1 cells (F4/80^+^CD11b^+^CD86^+^). **C:** The percentage of CD86 positive cells is shown in the bar graph. **D:** Black box displays the F4/80^+^CD11b^+^CD206^+ ^M2 macrophages. **E:** The percentage of CD206 positive cells is shown in the bar graph. *p < 0.01 compared to the DMEM group.

Flow cytometry was used to detect the effects of rTsgal and GM on macrophage polarization. The results showed that the percentage of M1 macrophages in rTsgal and GM treated-RAW264.7 cells was significantly increased compared to the DMEM control group (*F* = 43.66, *p *< 0.0001). Meanwhile, the percentage of M2 macrophages in the rTsgal and GM-treated group was not obviously increased compared to the DMEM group (Fig. 9). The M1 percentage of mouse peritoneal macrophages were also increased significantly in the rTsgal and GM group relative to the DMEM control group (*F* = 10.816, *p* = 0.002), whereas the percentage of M2 macrophages was not obviously increased (Fig. 10). There was no obvious difference in the polarization of RAW264.7 and peritoneal macrophages between the rTsgal and GM treatment groups. The results demonstrated that rTsgal and GM induced the activation of macrophages towards M1 polarization and evidently increased the percentage of CD86 positive cells.

### The killing effect of rTsgal and GM-treated peritoneal macrophage-mediated ADCC on NBL

**Figure 11:**
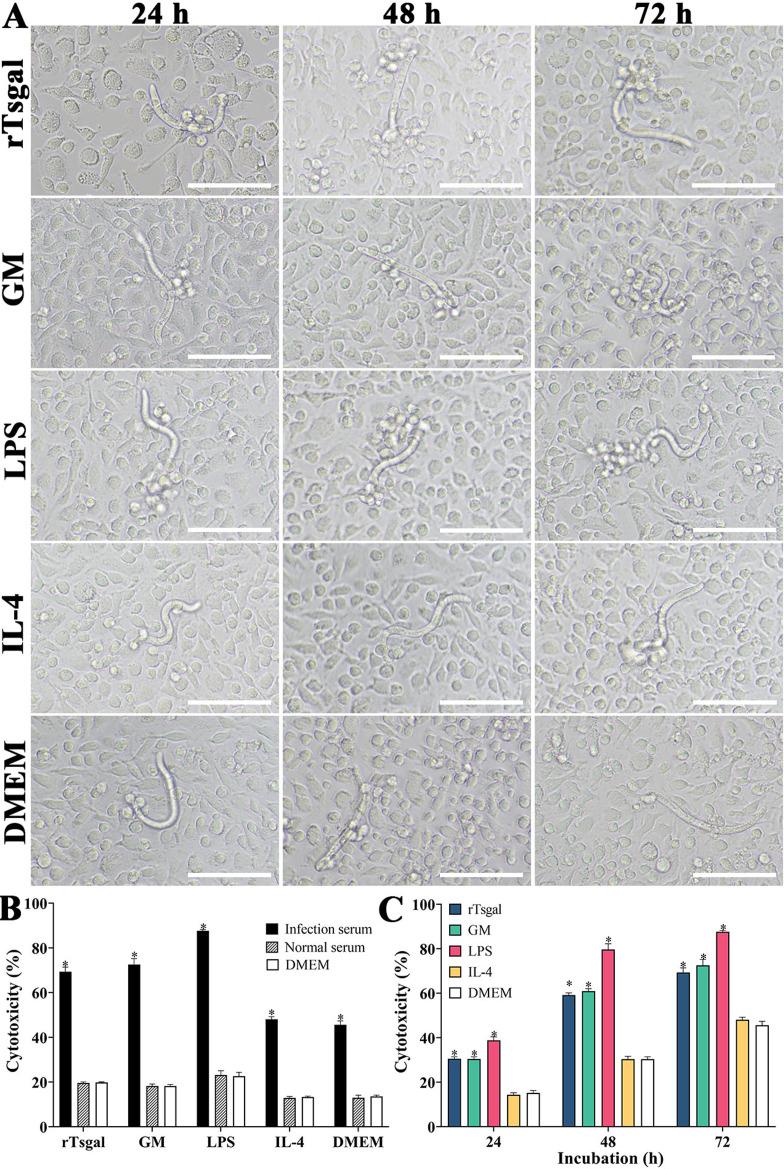
*Killing effects of ADCC on the NBL*. Murine peritoneal macrophages were pre-incubated with GM and rTsgal, 50 NBL were added into the medium containing infection serum and incubated at 37 °C for 72 h. After the NBL were cultured with various groups of macrophages and infection serum, larval viability was determined based on larval morphology and activity. Cytotoxicity was assessed as the percentage of dead NBL or NBL with adherent macrophages to the number of total larvae observed in each assay. **A:** The killing effect of ADCC on NBL was observed under microscopy at various cultivation times. **B:** The cytotoxicity of various groups of murine peritoneal macrophages on NBL at 72 h after cultivation. **C:** The cytotoxicity had an elevating trend with prolongation of culture time. *p < 0.05 compared to the DMEM group. Scale bars: 200 μm.

To evaluate the cytotoxicity of rTsgal- and GM-treated peritoneal macrophages on the NBL, an ADCC assay was carried out. The ADCC results showed that after culture at 37 °C for 24, 48 and 72 h, various sera mediated peritoneal macrophage adhesion to the NBL and NBL damage. In the groups of rTsgal- or GM treated-macrophages, cytotoxicity was evidently enhanced and more macrophages attached to the NBL (*F*_24h_ = 94.773, *F*_48h_ = 192.008, *F*_72h_ = 99.994, *p *< 0.0001). However, in the IL-4 and DMEM groups, the NBL had stronger motile ability, and fewer macrophages adhered to the NBL (Fig. 11). The results suggest that rTsgal and GM enhanced the killing effect of macrophage-mediated ADCC on NBL.

## Discussion

The activity of some polysaccharides as biological response modifiers has been widely studied [31]. Polysaccharides obtained from different sources have been reported to have anti-viral, anti-bacterial, anti-fungal, anti-parasite, and anti-tumor activities [12, 79]. Galactomannan is an important polysaccharide with diverse biological activities. A galactomannan isolated from seeds of *Mimosa scabrella* and its oxovanadium were found to have leishmanicidal activity [1]. However, the biological activity of galactomannan in anti-*Trichinella* infection is unknown. Therefore, the aim of this study was mainly to investigate the role of galactomannan against *T. spiralis* infection.

Our qPCR and Western blot results revealed that Tsgal was expressed in all the *T. spiralis* life-cycle phases, and the expression level of Tsgal in the intestinal stages (6 h IIL and 6 d AW) was obviously higher than the other stages, suggesting that Tsgal is an intestinal invasive protein that had direct contact with the IECs, and mediated IIL invasion of enteric epithelium [35, 65]. Additionally, oral administration of lactose alone significantly reduced intestinal AW burdens, suggesting that binding of Tsgal on the IIL with its receptors on the IECs could be blocked by lactose due to Tsgal containing two CRD structures [62, 64]. The results indicate that Tsgal plays a crucial role in *T. spiralis* invasion and might be a therapeutic target against *T. spiralis* invasion and infection. However, lactose could not increase the levels of host antibody and cellular immunity in mice. Therefore, appropriate carbohydrates as an adjuvant therapeutic agent for both impeding larval invasion and enhancing host immune response needed to be further screened and identified.

The ELISA results showed evident binding of galactomannan to rTsgal. Binding was dose-dependent for both galactomannan and rTsgal. The specific binding of galactomannan to rTsgal is likely related to Tsgal CRD structures, consequently abrogating rTsgal’s facilitating role on larval intrusion of IECs [20]. Molecularly, galactomannans are polysaccharides consisting of a mannose backbone with galactose side residues [48], may be recognized and combined in the CRD of rTsgal. Binding of galactomannan with rTsgal CRD might be suppressed and block the binding and interaction between galectin and its receptors in gut epithelium. Consequently galactomannan significantly impeded IIL invasion of IECs. The IIL intrusion of gut mucosa is the first crucial step for *T. spiralis* to infect the host in the life cycle [45]. The results of the *in vitro* IIL invasive test showed that rTsgal clearly facilitated the larval invasion of IECs, whereas galactomannan evidently inhibited larval invasion. The CRD of the amoebic galectin binds to TLR-2 and TLR-4 in human colonic cells, activating the “classic” signaling pathway of these receptors, and increasing pro-inflammatory cytokine expression, enhancing adhesion of trophozoites to the cells and cell damage [14]. rTsgal facilitation of larval invasion may be involved in the binding of rTsgal CRD to the TLRs in the IECs. Galactomannan inhibition on larval invasion is likely because the binding between galactomannan and Tsgal CRD on the surface of IILs competitively suppresses the binding of Tsgal to the TLRs (TLR-2/TLR-4) in IECs. However, the mechanism of galactomannan driving macrophage M1 polarization is so far unclear. Studies are needed to investigate which kinds of receptors Tsgal binds to (e.g., TLR2/TLR4) in macrophages and IECs by using GST pull-down, immune-precipitation, and mass spectrometry [52].

In helminth infection, macrophages orchestrate Th1/Th2 responses by responding to different environmental signals [44]. Exposure to LPS polarizes macrophages into the M1 phenotype, which is associated with the expression of pro-inflammatory cytokines (TNF-α, IL-1β, and IL-6) [38]. The macrophages express surface pattern recognition receptors (PPRs), which are the targets of various ligands including some polysaccharides [67]. The ligand-bound receptor complexes can trigger production and release of a wide range of mediators, including NO and pro-inflammatory cytokines, such as TNF-α, IL-1β, and IL-6 [29, 75]. The mediators are believed to confer bactericidal and cytostatic activities to macrophages and play an important role in the recruitment and accessibility of other inflammatory cells to an area of injury [8]. In the present study, to evaluate the effects of galactomannan and rTsgal on the activation of macrophages, the effector molecules of M1/M2 macrophages were determined. The results revealed that rTsgal induced high expression of iNOS (M1 specific) in RAW264.7 macrophages. Recently, another study showed that the polysaccharide isolated from *Paecilomyces cicadae* promoted a dramatic increase in the release of pro-inflammatory factors (IL-1β, IL-6 and TNF-α) from bone marrow-derived macrophages [49]. Our results revealed that the transcription levels of M1 genes (IL-1β, IL-6 and TNF-α) and surface molecular CD86 were significantly increased in galactomannan- and rTsgal-treated RAW264.7 macrophages. The similar immune modulatory effects of galactomannan and rTsgal on macrophage polarization were also further confirmed in murine peritoneal macrophages. However, the protein expression levels of M1/M2-related cytokines should be further determined by using ELISA in future study. The results indicated that galactomannan or rTsgal directly converted macrophage to a M1 phenotype *in vitro*, which may be crucial to enhance the ability of macrophages to kill and damage the newborn larvae at early intestinal stage of *T. spiralis* infection. Additionally, the results of Western blot, qPCR, and flow cytometry showed that the capacity of RAW264.7 and murine peritoneal macrophages to be polarized into M1 or M2 types had no evident difference. Therefore, RAW264.7 or murine peritoneal macrophages were used in different experiments. As RAW264.7 macrophages are the cell line with the same phenotype that ensures the repeatability of the experiment, RAW264.7 were used for the CCK-8 and NO assay. Murine peritoneal macrophages are stable primary cells with more capacity to adhere to culture dish than RAW264.7. To avoid cell disturbance and separation caused by NBL movement, murine peritoneal macrophages were used in the ADCC assay. Furthermore, as shown in Figure 6, protein band patterns of Arg1 expressed in RAW264.7 and murine peritoneal macrophages were clearly different. Arg1 expressed in RAW264.7 was a monomer band, whereas Arg1 expressed in murine peritoneal macrophages was a dimer band. This difference is likely because Arg1 is presented in a homotrimer and has 3 isoforms produced by alternative splicing. The splicing patterns of Arg1 were different in macrophages of various origins. This resulted in the difference of band pattern of Arg1 in macrophages from diverse sources [73].

The enzyme iNOS produces NO through the conversion of L-arginine into citrulline by utilizing NADPH and oxygen [28]. β-glucan polysaccharides have been widely studied as macrophage activators and are considered to be potent stimulators of the NO pathway [75]. Other classes of polysaccharides are also able to increase the production of NO, reactive oxygen species (ROS), TNF-α, and IL-6 in RAW264.7 cells through the MAPKs and NF-κB pathways mediated by TLR2 and TLR4 [66]. In this study, the release of NO in galactomannan- and rTsgal-treated macrophages was also assayed. The results showed that both galactomannan and rTsgal increased NO synthesis and secretion due to their activating macrophage M1 polarization. NO was associated with resistance to apoptosis and immune escape [6]. The larval killing capacity of galactomannan- and rTsgal-treated macrophages via ADCC was also significantly increased. Previous studies showed that macrophages directly killed and destroyed *T. spiralis* NBL by releasing NO and mediating ADCC as a kind of main effector cell [15]. These findings suggested that galactomannan and rTsgal enhanced the killing effect of macrophage-mediated ADCC on NBL by promoting macrophage M1 polarization, and M1 macrophages produced the natural killing molecules (NO) and secreted pro-inflammatory cytokines (TNF-α and IL-6). Galactomannan, as a polysaccharide containing mannose and galactose, not only blocked the interaction between rTsgal and its receptors through Tsgal CRD to inhibit larval invasion IECs, but also enhanced ADCC larvae killing via activating macrophages. Moreover, a recent study showed that vaccination of mice with rTsgal + GM elicited a Th1/Th2 immune response and protection, as demonstrated by significantly higher levels of serum anti-rTsgal antibodies, mucosal sIgA and cellular immune responses relative to individual rTsgal immunization. The mice treated with rTsgal + GM exhibited a 40.16 and 27.53% reduction of intestinal adult worms and muscle larvae following challenge. Galactomannan enhanced protective immunity, alleviated intestinal and muscle inflammation, and reduced worm burden of infected mice [74]. Therefore, galactomannan might be a major molecule regulating the interaction between *T. spiralis* and the IECs as well as macrophages, and modulating host’s immune responses. However, the molecular mechanism of galactomannan and rTsgal activation of macrophage M1 polarization is not clear; their corresponding receptors in macrophages and signaling pathways need to be identified and characterized in further studies.

In conclusion, galactomannan was able to bind to rTsgal, abrogate rTsgal facilitation of larval invasion of IECs *in vitro*, driven by macrophage M1 polarization, and enhanced the ADCC killing of NBL. Our results also showed that galactomannan and rTsgal triggered Th1 response in macrophages by releasing TNF-α, IL-1β, and IL-6. These pro-inflammatory cytokines are involved in the generation of NO and macrophage activation, which increased the killing roles of macrophages-mediated ADCC on NBL. However, the mechanism of galactomannan activation of macrophage M1 polarization needs to be investigated in further studies. The results suggested that Tsgal might be considered a potential vaccine target molecule against *T. spiralis* invasion, galactomannan might be regarded as a new adjuvant therapeutic agent to impede IIL intrusion at the early enteral stage of *Trichinella* infection, and also as a potential candidate adjuvant of anti-*Trichinella* vaccines to enhance host immune responses.

## References

[1] Adriazola IO, Evangelista Do Amaral A, Amorim JC, Correia BL, Petkowicz CL, Mercê AL, Noleto GR. 2014. Macrophage activation and leishmanicidal activity by galactomannan and its oxovanadium (IV/V) complex in vitro. Journal of Inorganic Biochemistry, 132, 45–51. 24169303 10.1016/j.jinorgbio.2013.09.017

[2] Ahmed SF, Oswald IP, Caspar P, Hieny S, Keefer L, Sher A, James SL. 1997. Developmental differences determine larval susceptibility to nitric oxide-mediated killing in a murine model of vaccination against *Schistosoma mansoni*. Infection and Immunity, 65, 219–226. 8975915 10.1128/iai.65.1.219-226.1997 PMC174579

[3] Amo-Aparicio J, Garcia-Garcia J, Francos-Quijorna I, Urpi A, Esteve-Codina A, Gut M, Quintana A, Lopez-Vales R. 2021. Interleukin-4 and interleukin-13 induce different metabolic profiles in microglia and macrophages that relate with divergent outcomes after spinal cord injury. Theranostics, 11, 9805–9820. 34815787 10.7150/thno.65203 PMC8581417

[4] Bai SJ, Han LL, Liu RD, Long SR, Zhang X, Cui J, Wang ZQ. 2022. Oral vaccination of mice with attenuated *Salmonella* encoding *Trichinella spiralis* calreticulin and serine protease 1.1 confers protective immunity in BALB/c mice. PLoS Neglected Tropical Diseases, 16, e0010929. 36445875 10.1371/journal.pntd.0010929 PMC9707759

[5] Bai Y, Ma KN, Sun XY, Dan Liu R, Long SR, Jiang P, Wang ZQ, Cui J. 2021. Molecular characterization of a novel cathepsin L from *Trichinella spiralis* and its participation in invasion, development and reproduction. Acta Tropica, 224, 106112. 34453915 10.1016/j.actatropica.2021.106112

[6] Bailey P, Chang DK, Forget MA, Lucas FA, Alvarez HA, Haymaker C, Chattopadhyay C, Kim SH, Ekmekcioglu S, Grimm EA, Biankin AV, Hwu P, Maitra A, Roszik J. 2016. Exploiting the neoantigen landscape for immunotherapy of pancreatic ductal adenocarcinoma. Scientific Reports, 6, 35848. 27762323 10.1038/srep35848 PMC5071896

[7] Bardi GT, Smith MA, Hood JL. 2018. Melanoma exosomes promote mixed M1 and M2 macrophage polarization. Cytokine, 105, 63–72. 29459345 10.1016/j.cyto.2018.02.002 PMC5857255

[8] Chang ZQ, Lee JS, Gebru E, Hong JH, Jung HK, Jo WS, Park SC. 2010. Mechanism of macrophage activation induced by beta-glucan produced from *Paenibacillus polymyxa* JB115. Biochemical and Biophysical Research Communications, 391, 1358–1362. 20026063 10.1016/j.bbrc.2009.12.064

[9] Chen F, Huang G. 2018. Preparation and immunological activity of polysaccharides and their derivatives. International Journal of Biological Macromolecules, 112, 211–216. 29382579 10.1016/j.ijbiomac.2018.01.169

[10] Cheng Y, Yu Y, Zhuang Q, Wang L, Zhan B, Du S, Liu Y, Huang J, Hao J, Zhu X. 2022. Bone erosion in inflammatory arthritis is attenuated by *Trichinella spiralis* through inhibiting M1 monocyte/macrophage polarization, iScience, 25, 103979. 10.1016/j.isci.2022.103979 PMC8914552 35281745

[11] Cui Z, Gong Y, Luo X, Zheng N, Tan S, Liu S, Li Y, Wang Q, Sun F, Hu M, Pan W, Yang X. 2023. β-Glucan alleviates goal-directed behavioral deficits in mice infected with *Toxoplasma gondii*. Parasites & Vectors, 16, 65. 36782332 10.1186/s13071-023-05686-4 PMC9926625

[12] Despommier DD. 1998. How does *Trichinella spiralis* make itself at home? Parasitology Today, 14, 318–323. 17040798 10.1016/s0169-4758(98)01287-3

[13] European Food Safety Authority; European Centre for Disease Prevention and Control. 2021. The European Union One Health 2019 zoonoses report. EFSA Journal, 19, e06406. 33680134 10.2903/j.efsa.2021.6406 PMC7913300

[14] Galván-Moroyoqui JM. 2011. Pathogenic bacteria prime the induction of Toll-like receptor signalling in human colonic cells by the Gal/GalNAc lectin carbohydrate recognition domain of *Entamoeba histolytica*. International Journal for Parasitology, 41, 1101–1112. 21787776 10.1016/j.ijpara.2011.06.003

[15] Gansmuller A, Anteunis A, Venturiello SM, Bruschi F, Binaghi RA. 1987. Antibody-dependent in-vitro cytotoxicity of newborn *Trichinella spiralis* larvae: nature of the cells involved. Parasite Immunology, 9, 281–292. 3601445 10.1111/j.1365-3024.1987.tb00508.x

[16] Gebreselassie NG, Moorhead AR, Fabre V, Gagliardo LF, Lee NA, Lee JJ, Appleton JA. 2012. Eosinophils preserve parasitic nematode larvae by regulating local immunity. Journal of Immunology, 188, 417–425. 10.4049/jimmunol.1101980 PMC3244516 22131328

[17] Guo KX, Bai Y, Ren HN, Sun XY, Song YY, Liu RD, Long SR, Zhang X, Jiang P, Wang ZQ, Cui J. 2020. Characterization of a *Trichinella spiralis* aminopeptidase and its participation in invasion, development and fecundity. Veterinary Research, 51, 78. 32539772 10.1186/s13567-020-00805-w PMC7296678

[18] Han C, Yu J, Zhang Z, Zhai P, Zhang Y, Meng S, Yu Y, Li X, Song M. 2019. Immunomodulatory effects of *Trichinella spiralis* excretory-secretory antigens on macrophages. Experimental Parasitology, 196, 68–72. 30316775 10.1016/j.exppara.2018.10.001

[19] Han Y, Yue X, Hu CX, Liu F, Liu RD, He MM, Long SR, Cui J, Wang ZQ. 2020. Interaction of a *Trichinella spiralis* cathepsin B with enterocytes promotes the larval intrusion into the cells. Research in Veterinary Science, 130, 110–117. 32171999 10.1016/j.rvsc.2020.03.012

[20] Hao HN, Lu QQ, Wang Z, Li YL, Long SR, Dan Liu R, Cui J, Wang ZQ. 2023. Mannose facilitates *Trichinella spiralis* expulsion from the gut and alleviates inflammation of intestines and muscles in mice. Acta Tropica, 241, 106897. 36931335 10.1016/j.actatropica.2023.106897

[21] Hao HN, Song YY, Ma KN, Wang BN, Long SR, Liu RD, Zhang X, Wang ZQ, Cui J. 2022. A novel C-type lectin from *Trichinella spiralis* mediates larval invasion of host intestinal epithelial cells. Veterinary Research, 53, 85. 36258242 10.1186/s13567-022-01104-2 PMC9580147

[22] Hu CX, Xu YXY, Hao HN, Liu RD, Jiang P, Long SR, Wang ZQ, Cui J. 2021. Oral vaccination with recombinant *Lactobacillus plantarum* encoding *Trichinella spiralis* inorganic pyrophosphatase elicited a protective immunity in BALB/c mice. PLoS Neglected Tropical Diseases, 15, e0009865. 34699522 10.1371/journal.pntd.0009865 PMC8547688

[23] Hu CX, Zeng J, Hao HN, Xu YXY, Liu F, Liu RD, Long SR, Wang ZQ, Cui J. 2021. Biological properties and roles of a *Trichinella spiralis* inorganic pyrophosphatase in molting and developmental process of intestinal larval stages. Veterinary Research, 52, 6. 33413587 10.1186/s13567-020-00877-8 PMC7791673

[24] Hu CX, Zeng J, Yang DQ, Yue X, Dan Liu R, Long SR, Zhang X, Jiang P, Cui J, Wang ZQ. 2020. Binding of elastase-1 and enterocytes facilitates *Trichinella spiralis* larval intrusion of the host’s intestinal epithelium. Acta Tropica, 211, 105592. 32565198 10.1016/j.actatropica.2020.105592

[25] Hu YY, Zhang R, Yan SW, Yue WW, Zhang JH, Liu RD, Long SR, Cui J, Wang ZQ. 2021. Characterization of a novel cysteine protease in *Trichinella spiralis* and its role in larval intrusion, development and fecundity. Veterinary Research, 52, 113. 34446106 10.1186/s13567-021-00983-1 PMC8390047

[26] Jhan MK, Chen CL, Shen TJ, Tseng PC, Wang YT, Satria RD, Yu CY, Lin CF. 2021. Polarization of type 1 macrophages is associated with the severity of viral encephalitis caused by Japanese encephalitis virus and dengue Virus. Cells, 10, 3181. 34831405 10.3390/cells10113181 PMC8621422

[27] Jin QW, Zhang NZ, Li WH, Qin HT, Liu YJ, Ohiolei JA, Niu DY, Yan HB, Li L, Jia WZ, Song MX, Fu BQ. 2020. *Trichinella spiralis* thioredoxin peroxidase 2 regulates protective Th2 immune response in mice by directly inducing alternatively activated macrophages. Frontiers in Immunology, 11, 2015. 33072069 10.3389/fimmu.2020.02015 PMC7544948

[28] Kashfi K, Kannikal J, Nath N. 2021. Macrophage reprogramming and cancer therapeutics: Role of iNOS-derived NO. Cells, 10, 3194. 34831416 10.3390/cells10113194 PMC8624911

[29] Kim HS, Kim YJ, Lee HK, Ryu HS, Kim JS, Yoon MJ, Kang JS, Hong JT, Kim Y, Han SB. 2012. Activation of macrophages by polysaccharide isolated from *Paecilomyces cicadae* through toll-like receptor 4. Food and Chemical Toxicology, 50, 3190–3197. 22687552 10.1016/j.fct.2012.05.051

[30] Lei JJ, Hu YY, Liu F, Yan SW, Liu RD, Long SR, Jiang P, Cui J, Wang ZQ. 2020. Molecular cloning and characterization of a novel peptidase from *Trichinella spiralis* and protective immunity elicited by the peptidase in BALB/c mice. Veterinary Research, 51, 111. 32891183 10.1186/s13567-020-00838-1 PMC7487599

[31] Leung MY, Liu C, Koon JC, Fung KP. 2006. Polysaccharide biological response modifiers. Immunology Letters, 105, 101–114. 16554097 10.1016/j.imlet.2006.01.009

[32] Li LG, Peng XC, Yu TT, Xu HZ, Han N, Yang XX, Li QR, Hu J, Liu B, Yang ZY, Xu X, Chen X, Wang MF, Li TF. 2022. Dihydroartemisinin remodels macrophage into an M1 phenotype via ferroptosis-mediated DNA damage. Frontiers in Pharmacology, 13, 949835. 36034842 10.3389/fphar.2022.949835 PMC9403990

[33] Li R, Li D, Wang H, Chen K, Wang S, Xu J, Ji P. 2022. Exosomes from adipose-derived stem cells regulate M1/M2 macrophage phenotypic polarization to promote bone healing via miR-451a/MIF. Stem Cell Research & Therapy, 13, 149. 35395782 10.1186/s13287-022-02823-1 PMC8994256

[34] Lima-Junior DS, Costa DL, Carregaro V, Cunha LD, Silva AL, Mineo TW, Gutierrez FR, Bellio M, Bortoluci KR, Flavell RA, Bozza MT, Silva JS, Zamboni DS. 2013. Inflammasome-derived IL-1β production induces nitric oxide-mediated resistance to *Leishmania*. Nature Medicine, 19, 909–915. 10.1038/nm.3221 23749230

[35] Liu RD, Cui J, Liu XL, Jiang P, Sun GG, Zhang X, Long SR, Wang L, Wang ZQ. 2015. Comparative proteomic analysis of surface proteins of *Trichinella spiralis* muscle larvae and intestinal infective larvae. Acta Tropica, 150, 79–86. 26184560 10.1016/j.actatropica.2015.07.002

[36] Liu RD, Wang ZQ, Wang L, Long SR, Ren HJ, Cui J. 2013. Analysis of differentially expressed genes of *Trichinella spiralis* larvae activated by bile and cultured with intestinal epithelial cells using real-time PCR. Parasitology Research, 112, 4113–4120. 24026388 10.1007/s00436-013-3602-1

[37] Manwarren T, Gagliardo L, Geyer J, Mcvay C, Pearce-Kelling S, Appleton J. 1997. Invasion of intestinal epithelia in vitro by the parasitic nematode *Trichinella spiralis*. Infection and Immunity, 65, 4806–4812. 9353069 10.1128/iai.65.11.4806-4812.1997 PMC175690

[38] Martinez FO, Gordon S. 2014. The M1 and M2 paradigm of macrophage activation: time for reassessment. F1000Prime Reports, 6, 13. 24669294 10.12703/P6-13 PMC3944738

[39] Perera N, Yang FL, Lu YT, Li LH, Hua KF, Wu SH. 2018. *Antrodia cinnamomea* galactomannan elicits immuno-stimulatory activity through Toll-like Receptor 4. International Journal of Biological Sciences, 14, 1378–1388. 30123083 10.7150/ijbs.24564 PMC6097488

[40] Ren HN, Bai SJ, Wang Z, Han LL, Yan SW, Jiang P, Zhang X, Wang ZQ, Cui J. 2021. A metalloproteinase Tsdpy31 from *Trichinella spiralis* participates in larval molting and development. International Journal of Biological Macromolecules, 192, 883–894. 34656542 10.1016/j.ijbiomac.2021.10.021

[41] Ren HN, Guo KX, Zhang Y, Sun GG, Liu RD, Jiang P, Zhang X, Wang L, Cui J, Wang ZQ. 2018. Molecular characterization of a 31 kDa protein from *Trichinella spiralis* and its induced immune protection in BALB/c mice. Parasites & Vectors, 11, 625. 30518426 10.1186/s13071-018-3198-5 PMC6282284

[42] Ren HN, Liu RD, Song YY, Zhuo TX, Guo KX, Zhang Y, Jiang P, Wang ZQ, Cui J. 2019. Label-free quantitative proteomic analysis of molting-related proteins of *Trichinella spiralis* intestinal infective larvae. Veterinary Research, 50, 70. 31547875 10.1186/s13567-019-0689-0 PMC6757440

[43] Ribicich MM, Fariña FA, Aronowicz T, Ercole ME, Bessi C, Winter M, Pasqualetti MI. 2021. Reprint of: A review on *Trichinella* infection in South America. Veterinary Parasitology, 297, 109540. 34384644 10.1016/j.vetpar.2021.109540

[44] Rolot M, Dewals BG. 2018. Macrophage activation and functions during helminth infection: Recent advances from the laboratory mouse. Journal of Immunology Research, 2018, 2790627. 30057915 10.1155/2018/2790627 PMC6051086

[45] Romarís F, Appleton JA. 2001. Invasion of epithelial cells by *Trichinella spiralis*: *in vitro* observations. Parasite, 8, S48–50. 11484381 10.1051/parasite/200108s2048

[46] Sehrawat S, Reddy PB, Rajasagi N, Suryawanshi A, Hirashima M, Rouse BT. 2010. Galectin-9/TIM-3 interaction regulates virus-specific primary and memory CD8 T cell response. PLoS Pathogens, 6, e1000882. 20463811 10.1371/journal.ppat.1000882 PMC2865527

[47] Song YY, Zhang Y, Ren HN, Sun GG, Qi X, Yang F, Jiang P, Zhang X, Cui J, Wang ZQ. 2018. Characterization of a serine protease inhibitor from *Trichinella spiralis* and its participation in larval invasion of host’s intestinal epithelial cells. Parasites & Vectors, 11, 499. 30189888 10.1186/s13071-018-3074-3 PMC6127903

[48] Srivastava M, Kapoor VP. 2005. Seed galactomannans: an overview. Chemistry & Biodiversity, 2, 295–317. 17191982 10.1002/cbdv.200590013

[49] Stenvinkel P, Ketteler M, Johnson RJ, Lindholm B, Pecoits-Filho R, Riella M, Heimbürger O, Cederholm T, Girndt M. 2005. IL-10, IL-6, and TNF-alpha: central factors in the altered cytokine network of uremia–the good, the bad, and the ugly. Kidney International, 67, 1216–1233. 15780075 10.1111/j.1523-1755.2005.00200.x

[50] Sun GG, Song YY, Jiang P, Ren HN, Yan SW, Han Y, Liu RD, Zhang X, Wang ZQ, Cui J. 2018. Characterization of a *Trichinella spiralis* putative serine protease. Study of its potential as sero-diagnostic tool. PLoS Neglected Tropical Diseases, 12, e0006485. 29758030 10.1371/journal.pntd.0006485 PMC5967804

[51] Sun Q, Huang J, Gu Y, Liu S, Zhu X. 2022. Dynamic changes of macrophage activation in mice infected with *Trichinella spiralis*. International Immunopharmacology, 108, 108716. 35344812 10.1016/j.intimp.2022.108716

[52] Sun R, Zhao X, Wang Z, Yang J, Zhao L, Zhan B, Zhu X. 2015. *Trichinella spiralis* paramyosin binds human complement C1q and inhibits classical complement activation. PLoS Neglected Tropical Diseases, 9, e0004310. 26720603 10.1371/journal.pntd.0004310 PMC4697845

[53] Sun X, Lv Z, Peng H, Fung M, Yang L, Yang J, Zheng H, Liang J, Wu Z. 2012. Effects of a recombinant schistosomal-derived anti-inflammatory molecular (rSj16) on the lipopolysaccharide (LPS)-induced activated RAW264.7. Parasitology Research, 110, 2429–2437. 22281546 10.1007/s00436-011-2782-9

[54] Tang S, Jiang M, Huang C, Lai C, Fan Y, Yong Q. 2018. Characterization of arabinogalactans from *Larix principis-rupprechtii* and their effects on NO production by macrophages. Carbohydrate Polymers, 200, 408–415. 30177181 10.1016/j.carbpol.2018.08.027

[55] Tao Y, Ma J, Huang C, Lai C, Ling Z, Yong Q. 2022. The immunomodulatory activity of degradation products of *Sesbania cannabina* galactomannan with different molecular weights. International Journal of Biological Macromolecules, 205, 530–538. 35217078 10.1016/j.ijbiomac.2022.02.122

[56] Tao Y, Wang T, Huang C, Lai C, Ling Z, Yong Q. 2021. Effects of seleno*-Sesbania canabina* galactomannan on anti-oxidative and immune function of macrophage. Carbohydrate Polymers, 261, 117833. 33766336 10.1016/j.carbpol.2021.117833

[57] Vasilev S, Mitic I, Mirilovic M, Plavsa D, Milakara E, Plavsic B, Sofronic-Milosavljevic L. 2023. *Trichinella* infection in Serbia from 2011 to 2020: a success story in the field of One Health. Epidemiology and Infection, 151, e20. 36655706 10.1017/S0950268823000109 PMC9990384

[58] Wang R, Zhang Y, Zhen J, Zhang J, Pang Z, Song X, Lin L, Sun F, Lu Y. 2022. Effects of exosomes derived from *Trichinella spiralis* infective larvae on intestinal epithelial barrier function. Veterinary Research, 53, 87. 36273217 10.1186/s13567-022-01108-y PMC9587624

[59] Wang Z, Hao C, Zhuang Q, Zhan B, Sun X, Huang J, Cheng Y, Zhu X. 2020. Excretory/Secretory products from *Trichinella spiralis* adult worms attenuated DSS-induced colitis in mice by driving PD-1-mediated M2 macrophage polarization. Frontiers in Immunology, 11, 563784. 33117347 10.3389/fimmu.2020.563784 PMC7575908

[60] Woods S, Schroeder J, Mcgachy HA, Plevin R, Roberts CW, Alexander J. 2013. MAP kinase phosphatase-2 plays a key role in the control of infection with *Toxoplasma gondii* by modulating iNOS and arginase-1 activities in mice. PLoS Pathogens, 9, e1003535. 23966857 10.1371/journal.ppat.1003535 PMC3744406

[61] Wu Z, Nagano I, Takahashi Y, Maekawa Y. 2016. Practical methods for collecting *Trichinella* parasites and their excretory-secretory products. Parasitology International, 65, 591–595. 27495839 10.1016/j.parint.2016.08.001

[62] Xu J, Yang F, Yang DQ, Jiang P, Liu RD, Zhang X, Cui J, Wang ZQ. 2018. Molecular characterization of *Trichinella spiralis* galectin and its participation in larval invasion of host’s intestinal epithelial cells. Veterinary Research, 49, 79. 30068382 10.1186/s13567-018-0573-3 PMC6071371

[63] Xu N, Bai X, Liu Y, Yang Y, Tang B, Shi HN, Vallee I, Boireau P, Liu X, Liu M. 2021. The anti-inflammatory immune response in early *Trichinella spiralis* intestinal infection depends on serine protease inhibitor-mediated alternative activation of macrophages. Journal of Immunology, 206, 963–977. 10.4049/jimmunol.2000290 PMC7887736 33495238

[64] Xu YXY, Zhang XZ, Weng MM, Cheng YK, Liu RD, Long SR, Wang ZQ, Cui J. 2022. Oral immunization of mice with recombinant *Lactobacillus plantarum* expressing a *Trichinella spiralis* galectin induces an immune protection against larval challenge. Parasites & Vectors, 15, 475. 36539832 10.1186/s13071-022-05597-w PMC9764493

[65] Yan SW, Hu YY, Song YY, Ren HN, Shen JM, Liu RD, Long SR, Jiang P, Cui J, Wang ZQ. 2021. Characterization of a *Trichinella spiralis* cathepsin X and its promotion for the larval invasion of mouse intestinal epithelial cells. Veterinary Parasitology, 297, 109160. 32522393 10.1016/j.vetpar.2020.109160

[66] Yang F, Li X, Yang Y, Ayivi-Tosuh SM, Wang F, Li H, Wang G. 2019. A polysaccharide isolated from the fruits of *Physalis alkekengi* L. induces RAW264.7 macrophages activation via TLR2 and TLR4-mediated MAPK and NF-κB signaling pathways. International Journal of Biological Macromolecules, 140, 895–906. 31442508 10.1016/j.ijbiomac.2019.08.174

[67] Yoon YD, Han SB, Kang JS, Lee CW, Park SK, Lee HS, Kang JS, Kim HM. 2003. Toll-like receptor 4-dependent activation of macrophages by polysaccharide isolated from the radix of *Platycodon grandiflorum*. International Immunopharmacology, 3, 1873–1882. 14636836 10.1016/j.intimp.2003.09.005

[68] Yu Q, Cheng P, Wu J, Guo C. 2021. PPARγ/NF-κB and TGF-β1/Smad pathway are involved in the anti-fibrotic effects of levo-tetrahydropalmatine on liver fibrosis. Journal of Cellular and Molecular Medicine, 25, 1645–1660. 33438347 10.1111/jcmm.16267 PMC7875896

[69] Yue WW, Yan SW, Zhang R, Cheng YK, Liu RD, Long SR, Zhang X, Wang ZQ, Cui J. 2022. Characterization of a novel pyruvate kinase from *Trichinella spiralis* and its participation in sugar metabolism, larval molting and development. PLoS Neglected Tropical Diseases, 16, e0010881. 36315477 10.1371/journal.pntd.0010881 PMC9621426

[70] Yue X, Sun XY, Liu F, Hu CX, Bai Y, Yang Q, Liu RD, Zhang X, Cui J, Wang ZQ. 2020. Molecular characterization of a *Trichinella spiralis* serine proteinase. Veterinary Research, 51, 125. 32988413 10.1186/s13567-020-00847-0 PMC7520982

[71] Zawistowska-Deniziak A, Bień-Kalinowska J, Basałaj K. 2021. Regulation of human THP-1 macrophage polarization by *Trichinella spiralis*. Parasitology Research, 120, 569–578. 33415398 10.1007/s00436-020-07000-y

[72] Zeng J, Zhang XZ, Zhang R, Yan SW, Song YY, Long SR, Dan Liu R, Wang ZQ, Cui J. 2021. Vaccination of mice with recombinant novel aminopeptidase P and cathepsin X alone or in combination induces protective immunity against *Trichinella spiralis* infection. Acta Tropica, 224, 106125. 34508714 10.1016/j.actatropica.2021.106125

[73] Zhang BC, Li Z, Xu W, Xiang CH, Ma YF. 2018. Luteolin alleviates NLRP3 inflammasome activation and directs macrophage polarization in lipopolysaccharide-stimulated RAW264.7 cells. American Journal of. Translational Research, 10(1), 265–273. PMC5801364 29423011

[74] Zhang R, Zhang XZ, Guo X, Han LL, Wang BN, Zhang X, Liu RD. 2023. The protective immunity induced by *Trichinella spiralis* galectin against larval challenge and the potential of galactomannan as a novel adjuvant. Research in Veterinary Science, 165, 105075. 37931574 10.1016/j.rvsc.2023.105075

[75] Zhang S, Zhang Q, Li C, Xing N, Zhou P, Jiao Y. 2023. A zinc-modified *Anemarrhena asphodeloides* polysaccharide complex enhances immune activity via the NF-κB and MAPK signaling pathways. International Journal of Biological Macromolecules, 249, 126017. 37517752 10.1016/j.ijbiomac.2023.126017

[76] Zhang XZ, Wang ZQ, Cui J. 2022. Epidemiology of trichinellosis in the People’s Republic of China during 2009–2020. Acta Tropica, 229, 106388. 35231417 10.1016/j.actatropica.2022.106388

[77] Zhang XZ, Yue WW, Bai SJ, Hao HN, Song YY, Long SR, Dan Liu R, Cui J, Wang ZQ. 2022. Oral immunization with attenuated *Salmonella* encoding an elastase elicits protective immunity against *Trichinella spiralis* infection. Acta Tropica, 226, 106263. 34879232 10.1016/j.actatropica.2021.106263

[78] Zheng L, Pan Y, Feng Y, Cui L, Cao Y. 2015. L-Arginine supplementation in mice enhances NO production in spleen cells and inhibits *Plasmodium yoelii* transmission in mosquitoes. Parasites & Vectors, 8, 326. 26070945 10.1186/s13071-015-0940-0 PMC4468801

[79] Zong A, Cao H, Wang F. 2012. Anticancer polysaccharides from natural resources: a review of recent research. Carbohydrate Polymers, 90, 1395–1410. 22944395 10.1016/j.carbpol.2012.07.026

